# Thermal Physical Property-Based Fusion of Geostationary Meteorological Satellite Visible and Infrared Channel Images

**DOI:** 10.3390/s140610187

**Published:** 2014-06-10

**Authors:** Lei Han, Lu Shi, Yiling Yang, Dalei Song

**Affiliations:** 1 School of Information Science and Engineering, Ocean University of China, 238 Songling Road, Qingdao 266100, China; E-Mails: lshilu2008@163.com (L.S.); yiling.yang@hotmail.com (Y.Y.); 2 State Key Laboratory of Severe Weather, Chinese Academy of Meteorological Sciences, Beijing 100081, China; 3 College of Engineering, Ocean University of China, 238 Songling Road, Qingdao 266100, China

**Keywords:** geostationary satellite, fusion, wavelet, infrared image

## Abstract

Geostationary meteorological satellite infrared (IR) channel data contain important spectral information for meteorological research and applications, but their spatial resolution is relatively low. The objective of this study is to obtain higher-resolution IR images. One common method of increasing resolution fuses the IR data with high-resolution visible (VIS) channel data. However, most existing image fusion methods focus only on visual performance, and often fail to take into account the thermal physical properties of the IR images. As a result, spectral distortion occurs frequently. To tackle this problem, we propose a thermal physical properties-based correction method for fusing geostationary meteorological satellite IR and VIS images. In our two-step process, the high-resolution structural features of the VIS image are first extracted and incorporated into the IR image using regular multi-resolution fusion approach, such as the multiwavelet analysis. This step significantly increases the visual details in the IR image, but fake thermal information may be included. Next, the Stefan-Boltzmann Law is applied to correct the distortion, to retain or recover the thermal infrared nature of the fused image. The results of both the qualitative and quantitative evaluation demonstrate that the proposed physical correction method both improves the spatial resolution and preserves the infrared thermal properties.

## 1. Introduction

The infrared (IR) channels of geostationary meteorological satellites contain important thermal information for meteorological research and applications. Raw IR data are often converted to brightness temperature, and are readily used in weather analysis, numerical weather prediction (NWP), and climate modeling. One limitation of IR data is the low spatial resolution. In contrast, the VIS channel has considerably higher resolution. The motivation for fusing the IR and VIS data is to produce data with improved spatial resolution.

Image fusion aims to integrate complementary information from multisensor data, such that the synthesized image is more suitable for human visual perception or further processing. Current image fusion methods are categorized into three levels: pixel, feature and decision [1,2]. Here, only pixel-level fusion is described. The simplest fusion method is based on pixel-wise comparisons, such as taking the maximum or average of the pixels of interest. Multi-resolution analysis [3,4] is a popular fusion approach. The Laplacian pyramid technique represents the first attempt to decompose and merge images hierarchically [5]. Another effective multi-resolution approach is the wavelet-based method [6–8]. After wavelet analysis, a wave of multi-resolution transform bases have been developed and applied to image fusion, including curvelet [9,10], contourlet [11], and nonsubsampled contourlet [12]. All of these methods aim to obtain better visual performance [13], but fail to take into account the underlying physical properties. Therefore, the fused image may suffer from spectral distortion despite the enhanced visual quality.

In addition to these general multi-resolution fusion methods, many techniques have been developed specifically for the fusion of multispectral satellite images. Some methods applied widely include the Hue-Saturation-Intensity (HSI) transform, the Brovey method, and Primary Component Analysis (PCA) [1]. Guo and Moore introduced the pixel block intensity modulation (PBIM) method [14] to modulate the high-resolution panchromatic band of the Landsat Thematic Mapper (TM) into its low-resolution thermal band, to increase the details in the thermal band. The enhanced smoothing filter-based intensity modulation (SFIM) method proposed by Liu is able to integrate lower resolution multispectral images with higher resolution panchromatic images [15]. Choi *et al.* applied a curvelet-based approach to fuse multispectral and panchromatic images [16]. Aanaes *et al.* proposed a solution to the ill-posed fusion problem by presenting a framework for pixel neighborhood regularization based on prior assumptions on the image data [17]. Despite their multispectral capability, these methods cannot be adapted directly to the fusion of VIS and IR images. Specifically, the PBIM method improves only the spatial topographical resolution, rather than the actual spectral resolution. It is not therefore applicable to situations where the image contrasts are produced by the terrain's thermal emission, such as clouds. Second, both the PBIM and SFIM methods are modeled on the terrain's reflective properties. However, in our problem, the infrared radiation is dominant. Moreover, all methods except the PBIM require that that source images must have overlapping spectral responses, such as those between RGB visible channels and the panchromatic channel. This requirement is not satisfied by the IR and VIS images.

Here we propose a novel thermal physical property-based post-correction solution to the fusion of geostationary satellite IR and VIS channel images. Unlike the fusion of multispectral and panchromatic images, for which multiple sources (R, G, B, and Pan) are required, our method requires only one VIS channel and one IR channel. By taking advantage of the infrared thermal properties, our objective is to generate a higher-resolution IR image from the VIS-IR composite image produced by regular fusion methods. The organization of this paper is as follows: in Section 2, we discuss the methodological details in two steps, namely multiwavelet fusion and the physical correction. Section 3 presents the experimental results and quantitative evaluations of the proposed approach. This is followed by a discussion and conclusions in Section 4.

## 2. Method

Given the VIS and IR satellite images, we produce a higher-resolution infrared image following a two-step procedure. To begin with, the low-resolution IR image is resampled and interpolated so that both images are on the same pixel scale. Then, we fuse the resampled IR image with the VIS image using a regular multi-resolution fusion algorithm, so that the texture details are extracted from the VIS image and transferred into the IR image. In this step, many aforementioned multi-resolution analysis techniques can satisfy our need. Here we choose a relatively new and mature method, the multiwavelet analysis. Second, based on the Stefan-Boltzmann Law, we convert the original IR image to a thermal radiation map, and use this map as a reference to perform a physical correction on the fused image. The purpose of this correction is to adjust the spectral distortions induced by the fusion. The details of the algorithm are as follows.

### 2.1. Multiwavelet Image Fusion

We adopt a multiwavelet image fusion algorithm to extract the fine textural features from the VIS image and impose them on the IR image. This multi-resolution approach enables the decomposition of the source images into multiple resolution scales such that the structural details of the VIS image are readily available. At this stage, the infrared thermal properties are not considered, and we merge the two images only at the pixel level. This leads to further thermal physical properties-based correction discussed in the next section. More information on the multiwavelet algorithm and its application to image fusion can be found in [18–20]. It should be noted that other multi-resolution image fusion methods can also be used in this step.

First, we apply the multiwavelet decomposition to the two source images. Their resultant coefficient arrays contain a low-frequency approximation component, or rough contrast, and multiple high-frequency detail components representing sharp edges and details at various scales. Fusion is achieved by recombining the coefficients from source images. In our case, we take the approximation coefficients of the IR image as the low frequency component of the fused image, so that substantial infrared properties are preserved. The high-frequency components are selected from either the IR or the VIS detail coefficients, depending on which displays more regional structural detail. This is implemented by comparing the regional variance of the two coefficient arrays. We take the coefficient with higher local variance, indicating more structural information. The purpose here is not only to bring in the richest structural detail, but also to take into account the surrounding regional landscape, to make a more reasonable selection. Mathematically, the local variance within an *N* × *N* window around a position (*x*, *y*) is defined as:

(1) $$\sigma^{2}\left(x,y\right)=\frac{1}{N^{2}}\sum_{i=1}^{N}\sum_{j=1}^{N}{\left[D\left(i,j\right)-\mu \right]}^{2}$$

(2) $$\mu (x,y)=\frac{1}{N^{2}}\sum_{i=1}^{N}\sum_{j=1}^{N}D\left(i,j\right)$$

where *D*(*i*, *j*) is the detail coefficient at (*i*, *j*), and *μ*(*x*, *y*) is the mean value in the window. The selection rule for the detail coefficients can thus be described as:

(3) $$\begin{array}{cc} D_{F}(x,y) & =\{\begin{array}{cc} D_{VIS}\left(x,y\right), & \sigma_{VIS}^{2}\left(x,y\right)\geq \sigma_{IR}^{2}\left(x,y\right)\\ D_{IR}\left(x,y\right), & \sigma_{VIS}^{2}\left(x,y\right)<\sigma_{IR}^{2}\left(x,y\right) \end{array} \end{array}$$

**Figure 1.**
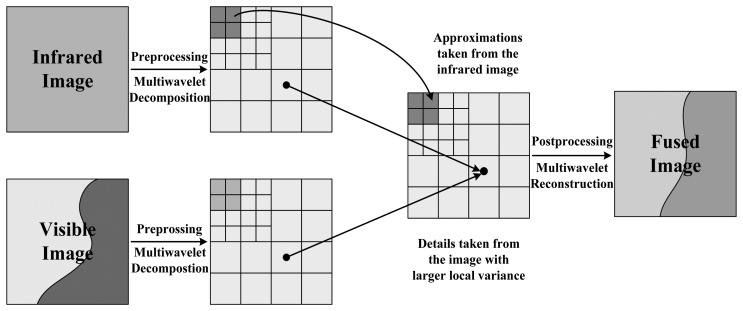
The multiwavelet-based algorithm for IR and VIS image fusion. Only three levels of decomposition are displayed.

An inverse multiwavelet transform of the selected approximation and detail coefficients recovers the fused image from the VIS and IR data. To sum up, this process can be summarized in the following steps, illustrated in Figure 1.

First, we pre-process the resampled IR image, and decompose both IR and VIS images using the CL multiwavelet system [21]. The selection of the number of decomposition levels is based on empirical outcome. Experiments show that six-level decomposition, or decomposition of the source images into six detail scales produces preferable fusion effects.

Second, we take the IR approximation coefficients as low-frequency components for the fused image, and use the local variance comparison method to select the high-frequency components. We use a 3 × 3 window to compare the local variance. Third, we apply the inverse multiwavelet transform on the selected coefficient arrays to produce the synthesized image from IR and VIS data.

### 2.2. Physical Property-Based Correction

The aforementioned multiwavelet-based method greatly enhances the visual resolution, but fails to preserve the infrared physical properties. Although the low-frequency components from the IR image remain untouched, the high-frequency information from the VIS image brings in serious spectral distortion. The distortion is particularly severe in areas where clouds are present only in the VIS image but it is spared in the IR image. After fusion, these areas may appear especially bright. This suggests a low brightness temperature if the infrared fusion outcome is assumed, which is not what the original IR image indicates. Therefore, a physical property-based post hoc correction is necessary to maintain or restore the infrared nature of the fused image.

The basic principle is to trace back to the fundamental property of the IR image, *i.e.*, the thermal radiation. Satellite infrared data are derived from the Earth's thermal radiation. This measured thermal radiation can be converted to brightness temperature. Inversely, given a brightness temperature, the original thermal radiation can be estimated. This temperature-to-radiation conversion can be approximated using the Stefan-Boltzmann Law, which is the integral of Planck's Law over the wavelengths and solid angles:

(4) $$j=ɛ\sigma T^{4}$$

Here *j* represents the total energy radiated per unit surface area per unit time, *T* is the temperature in Kelvin, *ε* is the emissivity and *σ* is the Stefan-Boltzmann constant. To dictate the infrared properties of the fused image (*i.e.*, to assume that the fused pixel values represent the brightness temperature), it is imperative that the underlying thermal radiation be comparable to that of the original IR image. The objective of the post hoc correction is therefore to constrain the regional radiation energy to the original IR level.

**Figure 2.**
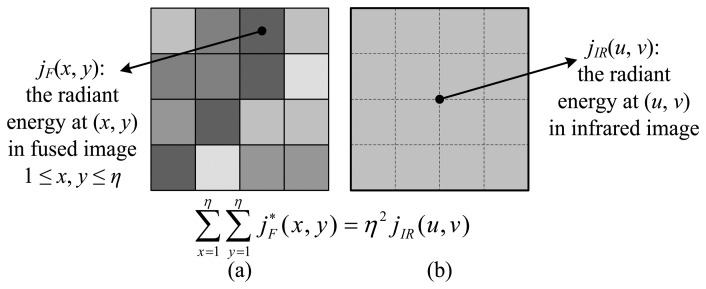
Illustration of physical correction. (**a**) A *η* × *η* window in the fused image. Here, *η* = 4. (**b**) One pixel in the low-resolution IR image. It is interpolated to the same scale as (a). Both windows in (a) and (b) should have the same amount of radiation energy.

Specifically, we first converted both the IR image and the synthesized fused image *F* into the radiation map, such that the values at each pixel, *j_IR_*(*u*, *v*) for IR image and *j_F_*(*x*, *y*) for the fused image, are the approximate amount of radiation energy observed at that pixel. Second, we traverse the two images and proportionally adjust the radiation energy in the fused image to ensure that the regional radiation is equal to the IR image within each window of interest. The choice of size of the window *η* takes the resolution disparity between the IR image and fused image (equal to the original VIS image) into account, such that one single pixel in the IR image corresponds to a *η* × *η* window in the fused image. This is essentially an interpolation process using the nearest-neighbor method (Figure 2). For instance, the satellite data have a VIS resolution of 1 km, and IR resolution of 4 km, so *η* is 4 in our case. Mathematically, letting j_F_*(*x*, *y*) be the corrected radiation energy at pixel (*x*, *y*), we expect that the following relation holds within each *η* × *η*:

(5) $$\sum_{x=1}^{\eta }\sum_{y=1}^{\eta }j_{F}^{*}\left(x,y\right)=\eta^{2}j_{IR}\left(u,v\right)$$

Accordingly, the old radiation value at pixel (*x*, *y*) in the fused image should be updated to:

(6) $$\begin{array}{cc} j_{F}^{*}\left(x,y\right)=j_{F}\left(x,y\right)\cdot \frac{\eta^{2}j_{IR}\left(u,v\right)}{\sum_{x=1}^{\eta }\sum_{y=1}^{\eta }j_{F}\left(x,y\right)}, & 1\leq x,y\leq \eta \end{array}$$

After the correction, the radiation maps are converted back to the brightness temperature.

**Figure 3.**
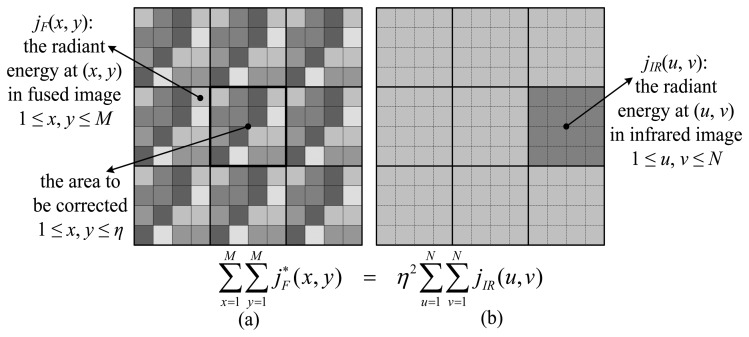
Improved physical correction. (**a**) An *M* × *M* area in the fused image is used to correct the highlighted *η* × *η* window in the center. (**b**) An *N* × *N* area in the low-resolution IR image. The objective is to let both areas in (a) and (b) have the same radiation energy.

However, a blocking result may occur due to the tight constraints. Because the process depends on only a single point in the IR image to correct a region, it fails to take into account the surrounding radiation landscape. If the values in one region need to be scaled upward while the adjacent window is scaled downward, for example, a clear border will be exposed. Consequently, the constraint needs to be loosened and more surrounding regions need to be included. Although a correction is still applied to each *η* × *η* window, we now consider a surrounding *N*-by-*N* area that is mapped to an *M*-by-*M* area in the IR image (Figure 3), instead of a single point, to decide the degree to which the radiation energy needs to be scaled. The *M*-to-*N* ratio is still kept equal as the ratio of the VIS and IR image resolution for computational ease. In this case, the equality of the regional radiation energy is expressed as:

(7) $$\sum_{x=1}^{M}\sum_{y=1}^{M}j_{F}^{*}\left(x,y\right)=\eta^{2}\sum_{u=1}^{N}\sum_{v=1}^{N}j_{IR}\left(u,v\right)$$

Accordingly, the corrected value in the fused image should be:

(8) $$\begin{array}{cc} j_{F}^{*}\left(x,y\right)=j_{F}\left(x,y\right)\cdot \frac{\eta^{2}\overset{N}{\underset{u=1}{\text{∑}}}\overset{N}{\underset{v=1}{\text{∑}}}j_{IR}\left(u,v\right)}{\overset{M}{\underset{x=1}{\text{∑}}}\overset{M}{\underset{y=1}{\text{∑}}}j_{F}\left(x,y\right)}, & 1\leq x,y\leq \eta \end{array}$$

## 3. Experimental Results and Analysis

We tested the proposed algorithm using the data collected by the Multifunctional Transport Satellite (MTSAT), a geostationary satellite operated by the Japan Meteorological Agency (JMA). It has five spectral bands: one visible channel (VIS) with 1-km resolution, and four IR channels (IR1∼4) with 4-km resolution. The VIS (0.55–0.90 μm) and IR1 (10.3–11.3 μm) channels were used to test the fusion algorithm. In this section, we first present three case studies to evaluate the algorithm based on visual inspection. Then, we introduce several objective measurements to quantitatively assess its performance.

### 3.1. Case Studies

**Figure 4.**
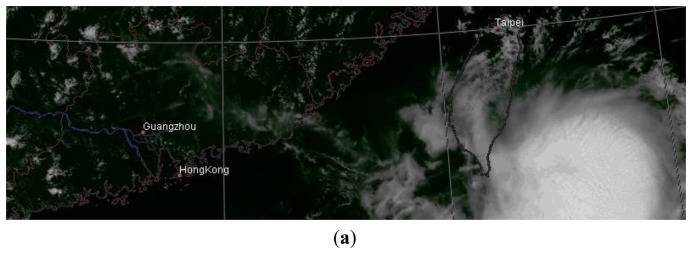

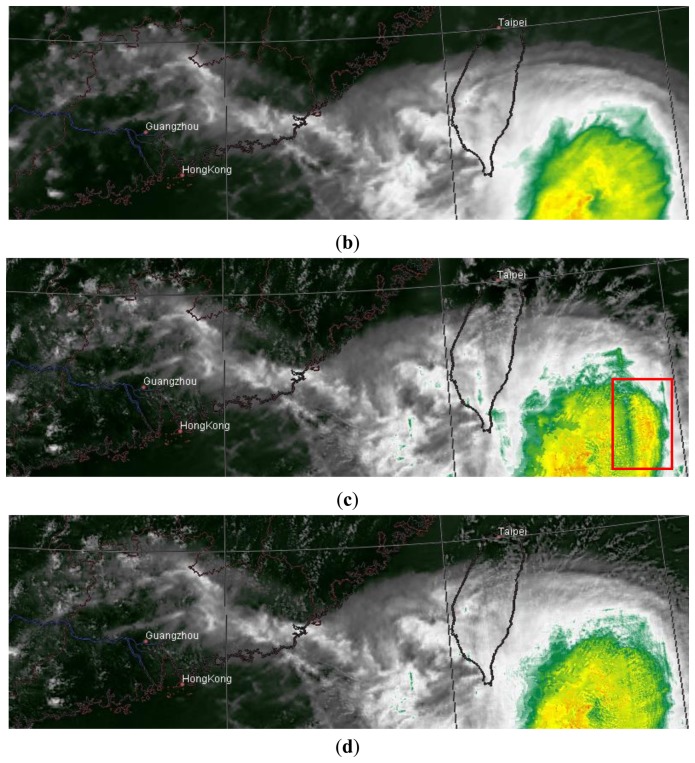
MTSAT images of South China and Taiwan on 1300 LST 24 July 2006. (**a**) VIS image. It fails to capture the peripheral clouds of the cyclone. (**b**) IR image. It presents the peripheral clouds that are absent in (a). (**c**) Fused image. It has higher resolution, and retains the cyclone peripheral clouds as in (b). The red box indicates an area with abnormal temperature. (**d**) Corrected image.

The first case (Figure 4) shows a cyclone near the South China coast and Taiwan on 1300 LST 24 July 2006. In Figure 4a, the VIS channel fails to capture some peripheral clouds of the cyclone that are present in the IR1 channel in Figure 4b. The multiwavelet fusion retains this feature of the IR1 image, and increases the overall image resolution, as shown in Figure 4c. However, as indicated in the red box, without a well-defined physical spectral characterization direct fusion incurs spectral distortion, exhibited by a prominent low temperature region. After the physical correction, the underlying pseudo-radiation level of the fused image is confined to the IR level, and therefore its infrared physical identity is established. As shown in Figure 4d, the corrected image has eliminated the abnormal temperature caused by spectral distortion, while the overall image maintains a higher resolution than the original IR1 image.

**Figure 5.**
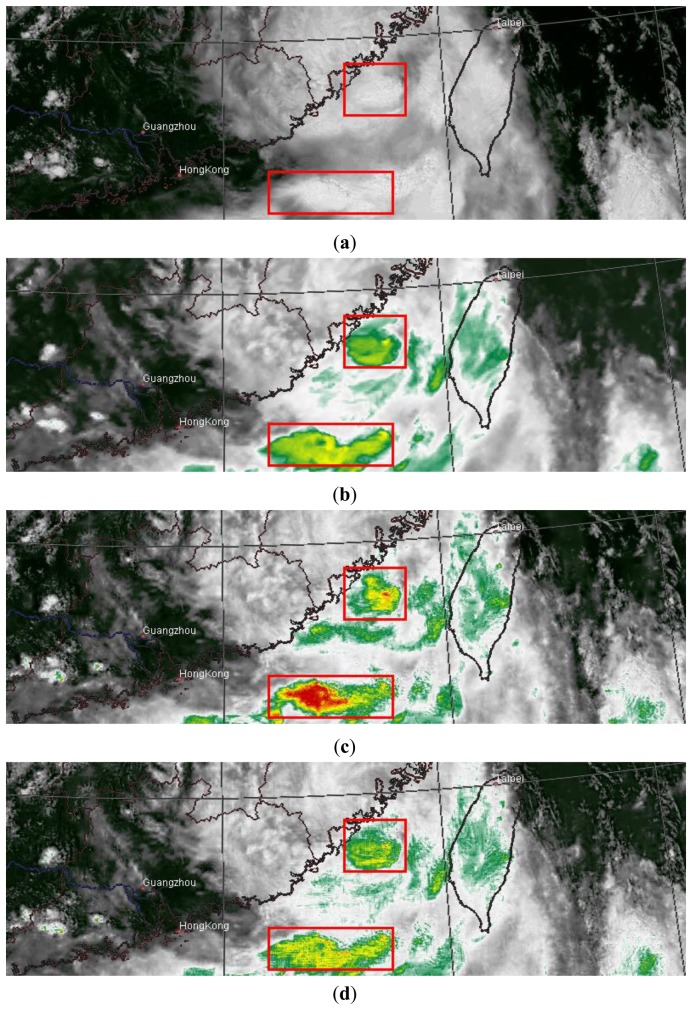
MTSAT images of South China and Taiwan on 1300 LST 25 July 2006. (**a**) VIS image; (**b**) IR image; (**c**) Fused image. Red boxes indicate areas with serious spectral distortion; (**d**) Corrected image.

The second case (Figure 5) was taken from the same region on 1300 LST 25 July 2006. The boxed regions illustrate how spectral distortion is incurred and corrected with our method. In the VIS image (Figure 5a), the red boxes enclose two regions with thick and bright cloud tops.

If these pixels are directly merged into the IR image (Figure 5b), unusually low temperatures will be induced, as illustrated in Figure 5c. The physical correction properly controls the level of distortion, and the abnormally low temperatures are recovered to the normal range (Figure 5d).

**Figure 6.**
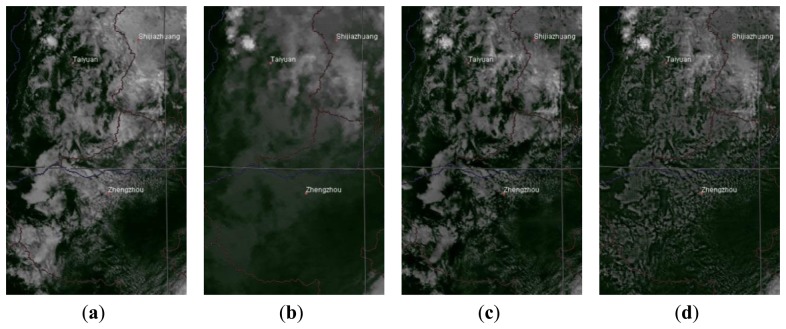
MTSAT image of the borders of Hebei, Shanxi and Henan Provinces of China (1300 LST 25 July 2006). (**a**) VIS image; (**b**) IR image; (**c**) Fused image; (**d**) Corrected image.

The third case (Figure 6) was taken from the border of China's Hebei, Shanxi and Henan Provinces on 1300 LST 25 July 2006. The VIS image (Figure 6a) indicates that most of the region is covered with scattered clouds, but the IR image (Figure 6b) suggests a relatively high brightness temperature in the entire region, and no obvious clouds are displayed. A direct fusion of VIS and IR images yields higher spatial resolution and incorporates fine cloud details from the VIS image. However, the overall temperature in the region is lowered significantly (Figure 6c). After the physical correction, the temperatures in this region are refined to the infrared level, as shown by the reduced brightness in Figure 6d, while the general cloud pattern from the VIS image is retained.

### 3.2. Quantitative Analyses

In addition to the visual inspection, we used the following five categories of parameters to objectively examine the performance of the fusion algorithm:

(1) Information Entropy (IE) and Mutual Information (MI). IE quantifies the amount of information contained within an image, and MI measures how much information is shared between images. These parameters can characterize the flow of information during the fusion process and the similarity of the synthesized and source images.
(2) Average Gradient (AG). The gradient at a pixel measures how sharply the pixel values change in the surrounding region. Fine details, sharp edges and complex textures produce high varying regional characteristics and are reflected by high gradients. The AG is the average of all regional gradients and reflects the overall sharpness of the image.
(3) Objective Fusion Performance Measure (Q*_abf_*). Proposed by Xydeas and Petrović [22], Q*_abf_* is a measurement of how much detailed edge information is transferred from the source images to the fused image. This parameter ranges between 0 and 1, where the value 0 represents the complete loss of edge information and 1 represents the perfect preservation of edges.
(4) Universal Image Quality Index (QI) and Edge-dependent Quality Index (QE). QI was initially proposed as a universal measure of image quality by modeling the structural distortion [23], but here we use it as an index of image similarity. This index does not depend on individual observers or testing conditions, and exhibits consistency with subjective evaluations. The dynamic range of QI is [−1, 1] [23]. The closer QI is to 1, the more similar the two images are in comparison. QE is adapted from QI such that edge information is taken into account [24]. In addition to the QI of the original images, QE also incorporates the QI of the corresponding edge images obtained from source images. Similarly, the values of QE still range between −1 and 1, with the best value of 1 achieved by perfect image similarity.
(5) Thermal Energy Deviation (AVGD and RMSD). Because the fusion method in this paper is based on thermal radiation, we introduce two new parameters: the average thermal energy deviation (AVGD), and the root-mean-square thermal energy deviation (RMSD). These allow us to evaluate the deviation of thermal energy between the synthesized image and the original IR image.

**Figure 7.**
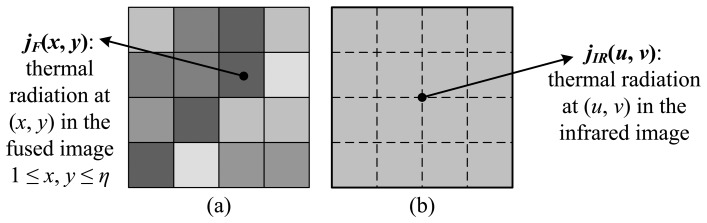
Illustration of the thermal energy deviation. (**a**) A *η* × *η* window in the fused image. In this case, *η* = 4. (**b**) One pixel in the low-resolution IR image. It is interpolated to the same scale as (a). The thermal energy deviation characterizes the difference of radiation energy in these *η* × *η* areas between fused image and IR image.

Assuming that the VIS-to-IR resolution ratio is *η*, a single pixel at (*u*, *v*) in the IR image corresponds to a *η* × *η* area in the fused image, which can be conceptualized as a simple interpolation (Figure 7). In each *η* × *η* window, the local thermal energy deviation from the original IR image is:

(9) $$\Delta j\left(u,v\right)=\sum_{x=1}^{\eta }\sum_{y=1}^{\eta }j_{F}\left(x,y\right)-\eta^{2}j_{IR}\left(u,v\right)$$

Traversing the calculation over the entire image, we have the AVGD, defined as:

(10) $$j_{AVGD}=\frac{1}{N^{2}}\sum_{u=1}^{N}\sum_{v=1}^{N}\left|\Delta j\left(u,v\right)\right|$$

Similarly, the RMSD is defined as:

(11) $$j_{RMSD}=\sqrt{\frac{1}{N^{2}}\sum_{u=1}^{N}\sum_{v=1}^{N}\Delta j{\left(u,v\right)}^{2}}$$

The purpose of the quantitative analysis is to evaluate the changes at the physical level. However, to the best of our knowledge, few metrics in the literature measure the similarity of the images' underlying thermal properties. Most of the metrics of choice here, e.g., AG and Qabf, are quality measures at the image level, so they may not perfectly satisfy our need. Fortunately, the fundamental physical-level similarity often externalizes at the image level, as demonstrated in the qualitative case studies in the previous session. Therefore, these image-level metrics may be of indirect interest to a certain extent. We have also customized two metrics, AVGD and RMSD, to compare the images' thermal properties directly. Moreover, it should be noted that what we evaluate is the relative change before and after the physical correction, instead of the simple absolute similarity with the original IR image. This reflects the compromise and trade-off between visual resolution and physical reality.

We arbitrarily selected the MTSAT data collected on 1300 LST 26 July 2006 to apply the multiwavelet fusion and physical correction, and calculated these performance parameters. The results are shown in Tables 1 and 2.

As shown in Table 1, the IE of the fused and corrected images are considerably higher than those of the original VIS and IR images, suggesting that the fusion process has indeed managed to integrate information from the two sources. Despite a slight loss of IE after the physical correction (from FUS to COR, about −0.9%), a significant amount is preserved. The MI values in Table 2 further reflect the “trade-off” and movement of information during the physical correction. The MI between COR and IR is about 33.8% higher than that between FUS and IR, yet a decrease of 6.1% is observed from FUS to COR with regard to the VIS image. This shift indicates that the physical correction obtains more meaningful information from the IR image while abandoning less relevant information from the VIS image. Notably, the 33.8% increase is greater than the 6.1% decrease, which means that much information from the VIS image is retained. Furthermore, with regard to the AG, both the FUS and COR images have higher gradients and thus higher spatial edges and details than the original IR image. This is consistent with subjective perception. The increase of AG from FUS to COR may be attributed to the disturbance caused by the introduction of noise.

In Table 2, both the FUS and COR images have higher QI with VIS images than with the IR image. The same trend is also observed for the QE parameter. The fusion result was closer to the VIS image than the IR image, indirectly suggesting increased spatial resolution after both the multiwavelet fusion and physical correction. Besides, by comparing the results before and after the physical correction; *i.e.*, FUS and COR, we noticed that the QI and QE increased from FUS to COR in the IR, while it decreased in the VIS. This again was due to the information trade-off in the physical correction, reflecting a gain in the meaningful thermal physical properties from the IR image and the loss of inaccurate visual information from the VIS image.

From the perspective of thermal radiation, as shown in Table 1, compared with the FUS image, the COR image exhibited much lower AVGD and RMSD (43.8% and 39.7% less, respectively), indicating that after the physical correction, the COR image deviated much less from the original IR image in terms of the underlying thermal radiation than the FUS image pre-correction. This was not surprising, because the physical correction process referred to the IR thermal radiation level to constrain that of the fused image, such that the outcome resembled the infrared physical properties. However, the cost was a slight loss of spatial resolution, especially the fine edge information, as reflected by the 13.5% decrease in Q*_abf_* in Table 1.

Fortunately, the case studies have shown that such a loss is not catastrophic, and the final corrected image still has high resolution compared with the original IR image. This is consistent with our fusion objective, where the re-establishment of physical truth is the primary goal, instead of visual sharpness. Tables 3, 4, 5 and 6 present similar results using data obtained on different days.

## 4. Conclusions

We propose a thermal physical properties-based correction method for fusing geostationary meteorological satellite IR and VIS images. Most existing image fusion techniques are capable of synthesizing multiple source images and producing satisfactory visual effects, but often fail to take into account the thermal physical properties of the IR images leading to spectral distortion. Our method mitigates the spectral distortion and reinstates the infrared physical properties of the fused image. We first apply a well-developed multiwavelet-based fusion method to merge the VIS and IR images, so that the rich textural details from the VIS image are used to increase the spatial resolution. Then, we use the thermal radiation inferred from the IR image as a reference to correct the fused image, to maintain a similar level of thermal radiation. The experimental results have demonstrated that the proposed method both improves the IR spatial property and preserves its spectral consistency.

The proposed fusion method has one limitation. This method is sensitive to the altitude of the sun at the time of data acquisition, or more precisely, the solar elevation angle. The algorithm performs best on data collected between 1100 and 1300 LST. At other times, the oblique incidence of sunlight may lead to a degradation of the data quality in the visible channel, compromising the fusion result. It will therefore be necessary to improve the method in the future by incorporating the influence of the angle between the sun and the satellite.

## Figures and Tables

**Table 1.** Quantitative analysis of IR and VIS image fusion and physical correction. The satellite data were collected on 1300 LST 26 July 2006. IR: infrared image. VIS: visible image. FUS: fused image using multiwavelet. COR: corrected image.

	**IE**	**AG**	**Q*_abf_***	**AVGD**	**RMSD**
**IR**	6.9091	0.6502			
**VIS**	4.9791	2.4797			
**FUS**	7.0560	2.7412	0.6510	667.0806	924.3553
**COR**	6.9890	2.8865	0.5633	375.2017	557.3362

**Table 2.** Quantitative analysis of IR and VIS image fusion and physical correction. The satellite data were collected on 1300 LST 26 July 2006.

	**MI**	**QI**	**QE**
**IR + FUS**	1.2882	0.1644	0.0867
**VIS + FUS**	0.8890	0.7895	0.7932
**IR + COR**	1.7235	0.1850	0.0893
**VIS + COR**	0.8350	0.7285	0.7169

**Table 3.** Quantitative analysis of IR and VIS image fusion and correction (1300 LST 24 July 2006).

	**IE**	**AG**	**Q*_abf_***	**AVGD**	**RMSD**
**IR**	6.9091	0.6502			
**VIS**	4.9791	2.4797			
**FUS**	7.0552	2.6771	0.6740	649.6730	906.2315
**COR**	6.9934	2.9388	0.5407	366.8883	551.5277

**Table 4.** Quantitative analysis of IR and VIS image fusion and correction (1300 LST 24 July 2006).

	**MI**	**QI**	**QE**
**IR + FUS**	1.3277	0.1829	0.0957
**VIS + FUS**	0.8830	0.8006	0.8114
**IR + COR**	1.7530	0.1936	0.1045
**VIS + COR**	0.8021	0.6969	0.6835

**Table 5.** Quantitative analysis of IR and VIS image fusion and correction (1300 LST 25 July 2006).

	**IE**	**AG**	**Q*_abf_***	**AVGD**	**RMSD**
**IR**	6.9567	0.6722			
**VIS**	5.0491	2.1981			
**FUS**	7.0574	2.4357	0.6451	578.3445	838.8759
**COR**	7.0288	2.6651	0.5244	336.5270	516.8839

**Table 6.** Quantitative analysis of IR and VIS image fusion and correction (1300 LST 25 July 2006).

	**MI**	**QI**	**QE**
**IR + FUS**	1.6075	0.2314	0.1405
**VIS + FUS**	0.9005	0.7447	0.7590
**IR + COR**	2.0161	0.2382	0.1278
**VIS + COR**	0.8724	0.6562	0.6478
